# Toxicokinetics, Percutaneous Absorption and Tissue Distribution of Benzophenone-3, an UV Filtering Agent, in Rats

**DOI:** 10.3390/toxics10110672

**Published:** 2022-11-07

**Authors:** Woohyung Jung, Su Hyun Seok, Soyoung Shin, Sung Ha Ryu, Kyu-Bong Kim, Beom Soo Shin, Tae Hwan Kim

**Affiliations:** 1 College of Pharmacy, Daegu Catholic University, Gyeongsan 38430, Gyeongbuk, Korea; 2 School of Pharmacy, Sungkyunkwan University, Suwon 16419, Gyeonggi, Korea; 3 College of Pharmacy, Wonkwang University, Iksan 54538, Jeonbuk, Korea; 4 College of Pharmacy, Dankook University, Cheonan 31116, Chungnam, Korea; 5 R&D Center, GL Pharm Tech Corp., Seongnam-si 13202, Gyeonggi, Korea; 6 Center for Human Risk Assessment, Dankook University, Cheonan 31116, Chungnam, Korea

**Keywords:** benzophenone-3, toxicokinetics, distribution, transdermal absorption, UV filtering agent

## Abstract

The aim of this study was to evaluate in vitro skin permeation and deposition, in vivo toxicokinetics, percutaneous absorption and tissue distribution of benzophenone-3 (BP-3) in rats. Four transdermal formulations containing BP-3 were prepared and evaluated for in vitro skin permeation and deposition of BP-3 using Franz diffusion cells. A gel formulation was used in subsequent in vivo percutaneous absorption due to its high in vitro skin permeation and deposition. Compared to intravenous (i.v.) injection, the prolonged terminal t_1/2_ (3.1 ± 1.6 h for i.v. injection and 18.3 ± 5.8 h for topical application) was observed indicating occurrence of flip-flop kinetics after topical application. The bioavailability of BP-3 after topical application was 6.9 ± 1.8%. The tissue-to-plasma partition coefficient (kp) for testis, considered a toxic target for BP-3, was less than 1. Overall, findings of this study may be useful for risk assessment of BP-3.

## 1. Introduction

Benzophenone-3 (2-hydroxy-4-methoxybenzophenone, BP-3), a UV filtering agent in cosmetic products and an organic ultraviolet (UV) filter in sunscreen that absorbs both UVa and UVb radiation at wavelengths from 200–350 nm, is widely used to protect the skin from UV rays [1,2,3,4]. Recently, as the use of sunscreens increases, the possibility of systemic exposure of BP-3 through the skin penetration is also increasing [5,6,7].

In many countries, the maximum allowable content of BP-3 in a product is regulated at 5–6% in the United States, Japan and Korea, and 10% in Europe [8,9,10,11].

To date, various studies about the toxicity of BP-3 including its endocrine disrupting potential have been reported [12,13,14,15,16]. By using MCF-7 and MDA-kb2-cells, BP-3 showed estrogenic and anti-androgenic effects. The pS2, estrogen-regulated protein was significantly up-regulated by BP-3 [17]. It is reported that BP-3 has both in vitro anti-estrogenic and anti-androgenic activities [18,19]. In addition, the mutagenic effect of BP-3 was observed in a salmonella assay [20], and the BP-3 induced sister chromatid exchange and chromosomal aberrations in Chinese hamster ovary cells [21].

Several reports about the in vivo endocrine disrupting activity of BP-3 have been published. Following oral and intra-peritoneal administrations, the increase of uterine weight was observed in rats [17,22,23]. In male rats, an antiestrogenic effect was also observed [24]. Furthermore, dietary contained 5% BP-3 decreased sperm concentration in caudal epididymis in F344/N rat and B6C3F mice [25].

The effect of formulation on in vitro skin permeation of BP-3 has been reported. Using a static diffusion cell, the permeability of BP-3 contained in emulsion and petroleum jelly was evaluated. The O/W emulsion maintained a high BP-3 concentration in the epidermis, dermis, stratum corneum, and receptor fluid, and showed a significant difference in increased permeability compared to the petroleum jelly [26]. Another study reported a significant difference in permeability and flux of BP-3 among light liquid paraffin, coconut oil, 50:50 ethanol:coconut oil, aqueous cream, and oily cream formulation. The highest flux was observed following the administration of light liquid paraffin [27].

Following a topical application of a cosmetic product containing BP-3, the systemic exposure of BP-3 was observed in human volunteers. The lotion containing 6% of BP-3 was applied and urine was collected for 48 h. Up to 48 h, BP-3 corresponding to 1-2% of the dose was detected in urine, which indicated a transdermal absorption of BP-3 [28]. BP-3 was also detected in urine after transdermal administration of sunscreen containing 4% BP-3 at 2 mg/cm^2^ in 11 volunteers. It is reported that the average total amount excreted was 11 mg, which is about 0.4% of the applied dose [29]. In a follow-up study, BP-3 was detected in urine after repeated transdermal administration of sunscreen containing 4% BP-3 for 5 days on the whole bodies of 25 volunteers. The excreted BP-3 via urine was 1.2–8.7% of the dosed amount, suggesting the potential for accumulation of BP-3 by daily exposure [5]. After repeated topical applications of 2 mg/cm^2^ sunscreen containing 10% BP-3, plasma and urine concentrations were determined. BP-3 was detected in plasma 1 h after the first administration [30]. Following lotion and aerosol spray containing BP-3 that were repeatedly applied to the skin for 4 days, BP-3 was detected in plasma. BP-3 was detected in the stratum corneum even on days 7 and 14 following the first administration [31,32]. To date, toxicokinetic parameters including absolute bioavailability in humans have not been reported.

In animal studies, BP-3 has been reported to be rapidly absorbed following oral and transdermal administration in rats and piglets [33,34,35,36]. Following oral administration, BP-3 concentration in the liver was highest among tissues [34]. Although various animal experiments have been reported, the absolute bioavailability and tissue distribution at the steady state of BP-3 have been limitedly identified.

Therefore, the present study was carried out to investigate the effect of the formulation on the skin permeability, the toxicokinetics including absolute bioavailability, and the tissue distribution at a steady state of BP-3.

## 2. Materials and Methods

### 2.1. Materials

The following materials were used as received, without further purification: petroleum jelly (Unilever, London, UK), medium-chain triglycerides (Sasol Germany GmbH, Hamburg, Germany), marketed body emulsion containing “free” BP-3 (Neutrogena Co., Los Angeles, CA, USA), carbomer 940 (Lubrizol Corp., Wickliffe, OH, USA), poloxamer 188 (BASF, Ludwigshafen, Germany). BP-3, nonivamide, acetic acid, formic acid, ethylenediamine tetraacetic acid (EDTA) sodium, methyl paraben, mineral oil, sodium hydroxide, and beeswax were purchased from Sigma Aldrich Co. (St. Louis, MO, USA). Acetonitrile, water, and methanol (all HPLC grade) were purchased from Avantor (Radnor, PA, USA).

### 2.2. HPLC-UV and LC-MS/MS Analysis

BP-3 concentrations in samples obtained from in vitro skin permeation studies were determined by a HPLC-UV. A Waters 2690 separation module with a Waters 2487 dual λ absorbance detector (Waters, Milford, MA, USA) was applied. The UV detection wavelength was set at 290 nm. The mobile phase was a mixture of acetonitrile and 0.1% acetic acid (70:30 *v*/*v*%), and the flow rate of the mobile phase was 1.2 mL/min. The lower limit of quantification (LLOQ) was 50 ng/mL. The receptor fluid samples were analyzed without extraction steps, and stripped tapes and harvested skin layers were extracted with methanol for 12 h. Separately, BP-3 concentrations in plasma, skin and tissue samples obtained from the in vivo toxicokinetic studies were determined by a validated liquid chromatography mass spectroscopy (LC-MS/MS) method. An API 2000 LC-MS/MS system (Applied Biosystems/MDS Sciex, Toronto, ON, Canada) coupled with a Waters 2690 HPLC (Waters Corporation, Milford, MA, USA) was applied. Rat biological samples were prepared by protein precipitation with acetonitrile. The 150 μL of acetonitrile was added to 50 μL of plasma sample. The mixture was vortex-mixed for 1 min and then centrifugated for 10 min at 4000× *g*. The 100 μL of supernatant was diluted with an identical volume of distilled water and a portion (10 μL) was injected into LC-MS/MS. For preparation of tissue homogenates samples, 350 μL of acetonitrile was added to 50 μL of biological samples. The subsequent procedure was the same as the plasma preparation. Washed swabs and stripped tapes were extracted with methanol with a 12-h agitation in a shaking bath (Lab Companion, Daejeon, Korea). Separations were achieved on a Kinetex C18 column (50 × 2.1 mm ID, 2.6 μm, Phenomenex, Torrance, CA, USA). The mobile phase was a mixture of methanol and 0.05% formic acid (85:15 *v*/*v*%), and the flow rate of the mobile phase was 0.3 mL/min. The multiple reaction monitoring was based on the transition of m/z of 229.0 → 150.9 for BP-3 and 294.3 → 137.2 for nonivamide (internal standard). The LLOQ of plasma and tissue homogenates were 1 and 5 ng/mL, respectively. The assay was validated using the matrix-matched quality control (QC) samples (including QC samples at LLOQ). The intra- and inter-day accuracy and precision ranged from 97.5% to 109.1% and from 1.3% to 8.5%, respectively.

### 2.3. In Vitro Skin Permeability

Rat skins were mounted on Franz diffusion cells (area: 1.77 cm^2^). The receptor cell (volume: 10 mL) was filled with phosphate buffered saline (PBS, pH 7.4) containing 5% Tween 80 to maintain sink conditions. After equilibration, approximately 200 mg of transdermal formulations (petroleum jelly, oil, lotion, and gel) with incorporated BP-3 were applied to the donor cell. The protocol for incorporation of BP-3 in transdermal formulations have been described previously [37]. The composition of BP-3 formulations was listed in Table 1. The receptor medium was stirred at 600 rpm and maintained at 37 ± 0.5 °C throughout the experiment for 48 h. At 0, 2, 4, 6, 8, 12, 24, 36, and 48 h following applications, receptor media were collected and replaced with an equal volume of fresh media. Immediately after the last receptor medium collection, the stratum corneum (30 times of tape strips), viable epidermis, and dermis were separated to determine amounts of BP-3 in the skin. The collected samples were kept at −20 °C until analysis.

### 2.4. In Vivo Toxicokinetics

To characterize the dermal absorption kinetics, BP-3 was intravenously and topically administered in male Sprague-Dawley rats (8–10 weeks of age) (Samtako Co., Osan, Gyeonggi-do, Korea). Prior to the study, the rats were acclimatized for at least 1 week at a temperature of 23 ± 2 °C with a 12-h light/dark cycle and a relative humidity of 50%. BP-3 was intravenously injected at a dose of 1 mg/kg. The dosing solutions were prepared by dissolving BP-3 in a mixture of ethanol:PEG 400:Tween 80:saline (2:2.5:0.5:5, *v*/*v*) at concentrations of 0.5 mg/mL. Blood samples were collected from the jugular vein at 0, 2, 5, 15, 30 min, 1, 2, 4, 8, and 12 h following injection. Plasma samples were harvested by centrifugation at 4000× *g* for 10 min and stored at −20 °C until analysis.

In vivo transdermal absorption kinetics of BP-3 were characterized after topical application of gel formulation. Prior to administration, the back skin of the rat covering an area of 3 × 3 cm was shaved with an electric clipper (Golden A5, Oster, Rye, NY, USA). Next, the 200 mg of BP-3 containing gel was evenly applied to the shaved area. At 0, 6, 12, 24, 36, 48, 72, and 96 h following application, blood samples were collected from the jugular vein and centrifuged at 4000× *g* for 10 min. Immediately after the last blood sample collection, the animals were sacrificed and the stratum corneum (30 times of tape strips), viable epidermis, and dermis were separated to determine amounts of BP-3 in the skin. The viable epidermis and dermis were homogenized in saline (Tissue tearer, Biospec Co., Bartlesville, OK, USA). The collected samples were stored at −20 °C until analysis.

The toxicokinetic parameters were calculated by standard noncompartmental analysis in WinNonlin (Pharsight, Cary, NC, USA). These parameters included the initial plasma concentration (C_0_), area under the plasma concentration–time curve from time zero to last sampling point (AUC_last_), and terminal half-life (t_1/2_). The peak plasma concentration (C_max_) and the time to reach C_max_ (T_max_) were directly obtained from the observations. The absolute bioavailability (F) of BP-3 after transdermal application was calculated as F (%) = 100 (AUC_transdermal_ × Dose_i.v._)/(AUC_i.v._ × Dose_transdermal_).

BP-3 tissue distribution at a steady state was evaluated following continuous i.v. infusion at a rate of 0.23 mg/h for 12 h. At 0, 2, 4, 8, 12 h following administration, blood samples were collected via the jugular vein and centrifuged at 4000× *g* for 10 min. Harvested plasma samples were immediately stored at −20 °C until analysis. At 12 h, kidney, lung, brain, heart, liver, spleen, testis, stomach, small intestine, and large intestine were collected. Collected samples were homogenized in saline. The tissues were stored at −20 °C until analysis. Using plasma and tissue concentration of BP-3 at steady state, the partition coefficient (k_p_) as the tissue-to-plasma concentration ratio for each tissue was calculated.

## 3. Results

### 3.1. In Vitro Skin Permeation of BP-3

**Figure 1:**
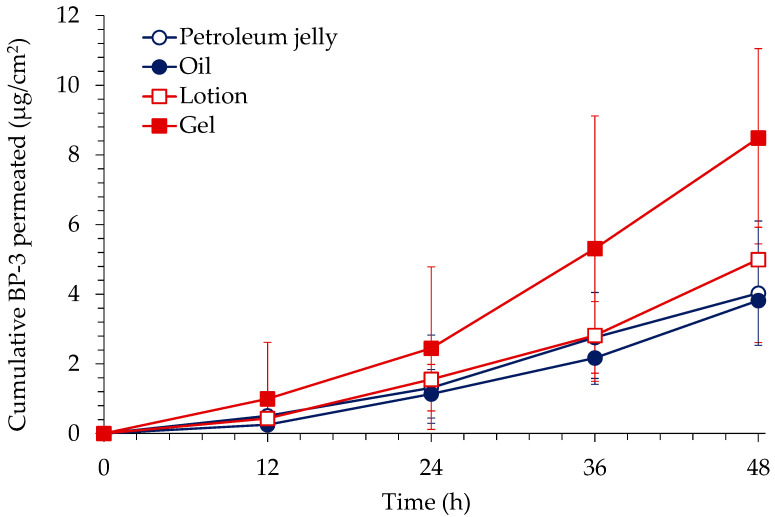
In vitro skin permeation profile of BP-3 through excised rat skin after application of BP-3 formulations (mean ± S.D., n = 6 each).

In vitro skin permeation profiles of BP-3 through excised rat skin after application of BP-3 formulations are presented in Figure 1. The respective flux of BP-3 was 0.11 ± 0.06 μg/cm^2^/h for petroleum jelly, 0.11 ± 0.05 μg/cm^2^/h for oil, 0.14 ± 0.03 μg/cm^2^/h for lotion, and 0.25 ± 0.02 μg/cm^2^/h for gel. The flux for gel formulation was significantly higher than that for the other formulations (*p* < 0.05, one-way ANOVA, post hoc; Tukey’s).

In vitro skin deposition of BP-3 in the stratum corneum and the epidermis/dermis of excised rat skin after 48 h application of BP-3 formulations is summarized in Table 2. Except for the gel, the deposition of BP-3 in the stratum corneum was similar to that in the epidermis/dermis. In the case of the gel, the deposition of BP-3 in the stratum corneum, which is considered to be able to be systemically exposed, was significantly higher than that in the epidermis/dermis.

Among the formulations tested for in vitro skin permeation and deposition of BP-3, gel formulation showed the highest skin permeation and deposition. Consequently, gel formulation was used for further in vivo toxicokinetic study.

### 3.2. In Vivo Percutaneous Absorption

**Figure 2:**
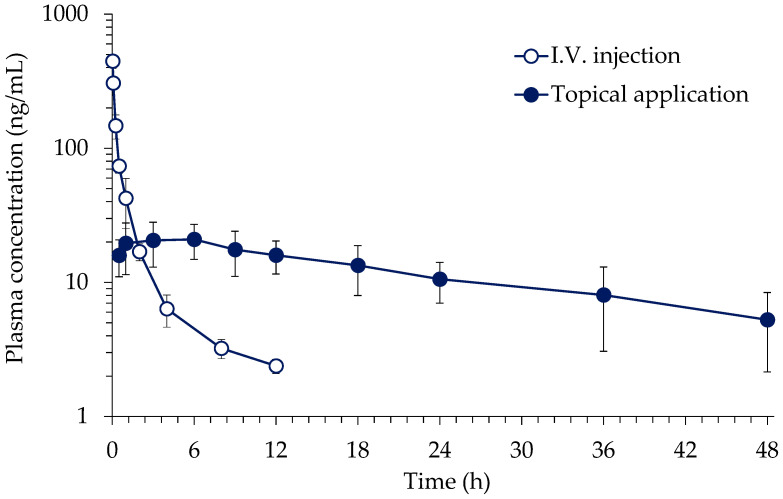
Average plasma concentration-time profiles of BP-3 obtained after i.v. injection and transdermal administration at doses of 1 mg/kg (n = 4) and 40 mg/kg (n = 5), respectively, in rats (mean ± S.D.).

This study was performed to characterize the disposition and topical bioavailability of BP-3. The average plasma concentration-time profiles of BP-3 obtained after i.v. injection (1 mg/kg) and topical application (40 mg/kg) are presented in Figure 2. A multi-exponential decline was observed in BP-3 plasma concentration after i.v. injection. Following topical application, BP-3 was slowly absorbed and eliminated.

The mean toxicokinetic parameters of BP-3 after i.v. injection and topical application in rats are summarized in Table 3. The t_1/2_ was significantly different between groups, indicating the occurrence of flip-flop kinetics after the topical application of gel. The mean bioavailability of BP-3 after topical application of the gel was 6.9%.

### 3.3. Tissue Distribution

**Figure 3:**
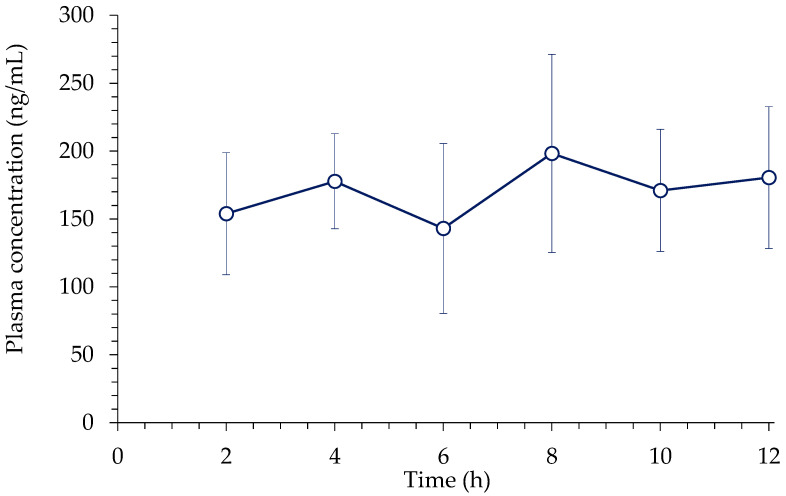
Average plasma concentration-time profile of BP-3 during i.v. infusion in rats (n = 4, mean ± S.D.).

Since BP-3 is considered to be exposed continuously, the tissue distribution of BP-3 was evaluated under a steady state. The average plasma concentration-time profile of BP-3 during i.v. infusion is shown in Figure 3. At 12 h following i.v. infusion, the average observed plasma concentration was 180.50 ± 52.29 ng/mL. The steady-state concentrations and k_p_ values of BP-3 in ten different tissues are summarized in Table 4. The highest k_p_ was observed for the large intestine, followed by the lung, kidney, small intestine, stomach, heart, brain, liver, spleen, and testis.

## 4. Discussion

Although BP-3, a representative UV filtering agent has endocrine disrupting potential, limited toxicokinetic properties for risk assessment have been reported to date. In the present study, we investigated the in vitro skin permeability of BP-3 using four different transdermal formulations, in vivo toxicokinetics and tissue distribution.

From the in vitro diffusion study, penetration rate of BP-3 from gel formulation was significantly higher than other formulations which indicates the presence of significant effect of the formulation on BP-3 skin penetration. According to OECD guidance, chemical in the stratum corneum remaining after topical exposure is considered to be systemically absorbable [38]. Following the gel administration, about 17% of dosed amount of BP-3 remained in the stratum corneum. Therefore, it was expected that a significant amount of BP-3 contained in the gel formulation would penetrate the skin.

To assume the worst-case scenario, the gel was applied to an in vivo toxicokinetic study. In this study, the absolute bioavailability of BP-3 following topical application was calculated as 6.9 ± 1.8%. The terminal t_1/2_ was significantly longer in the transdermal administration group than the i.v. injected group, which is suggestive of a flip-flop. A flip-flop is observed when the absorption rate from dosing area into the blood is significantly slower than the elimination rate of the chemical in the blood. In the present study, the slow absorption rate of BP-3 through skin layers was reflected on the elimination phase of the plasma concentration-time profile, making it seem like an increase of the half-life. Considering in vitro diffusion results, this flip-flop phenomenon is expected to have occurred by the sustained transdermal absorption of BP-3 from the stratum corneum. In the previous study, a similar flip-flop was observed following a gel formulation containing homosalate, an UV filtering agent [37].

Due to the flip-flop phenomenon during transdermal absorption, the duration of systemic exposure of BP-3 was significantly prolonged. As such, as the half-life is prolonged by flip-flop, the accumulation ratio and time to reach a steady state of the chemical following repeated exposure may significantly increase. Thus, in the case of the skin permeability and overall systemic absorption rate being affected by a change of formulation, the characterization of in vivo toxicokinetics becomes more important for accurate risk assessment.

Following i.v. infusion, BP-3 was mainly distributed to the large intestine, lung, and kidney. BP-3 distribution to the testis, considered as a toxic target due to the anti-androgenic and estrogenic effect of BP-3, was lower than that of most tissues.

## 5. Conclusions

Through this study, we suggested that the skin permeability of BP-3 is significantly affected by the formulation, and we investigated the overall toxicokinetic properties including absorption kinetics, disposition and tissue distribution. These results are expected to be utilized for the further risk assessment of BP-3.

## Figures and Tables

**Table 1:** Composition of BP-3 formulations.

Petroleum Jelly	Oil	Lotion	Gel
Benzophenone-3 (5%)	Benzophenone-3 (5%)	Benzophenone-3 (5%)	Benzophenone-3 (5%)
Petroleum jelly (95%)	Miglyol (18.7%)	Miglyol (18.7%)	Miglyol (18.7%)
	Mineral oil (72.3%)	Marketed body emulsion (75.8%)	Carbomer 940 (1.1%)
	Bees wax (4.0%)	Surfactant (0.5%)	Poloxamer 188 (0.2%)
			EDTA Na (0.1%)
			Methyl paraben (0.1%)
			NaOH (10.0%)
			Surfactant (0.5%)
			DW (64.3%)

**Table 2:** In vitro skin deposition of BP-3 in the stratum corneum and the epidermis/dermis of excised rat skin after 48 h application of BP-3 formulations (mean ± S.D., n = 6).

Formulation	Epidermis/Dermis (%)	Stratum Corneum (%)
Petroleum jelly	2.1 ± 0.3	1.0 ± 0.5
Oil	2.1 ± 0.6	2.8 ± 1.6
Lotion	3.3 ± 0.9	3.4 ± 1.3
Gel	4.7 ± 1.0	17.0 ± 4.5 *

* Gel was significantly different from petroleum jelly, oil, and lotion (one-way ANOVA, post hoc; Tukey’s test, *p* < 0.05).

**Table 3:** Toxicokinetic parameters of BP-3 obtained after i.v. injection and transdermal administration at doses of 1 mg/kg and 40 mg/kg, respectively, in rats (mean ± S.D.).

Parameters	i.v. (n = 4)	Transdermal (n = 5)
t_1/2_ (h) *	3.1 ± 1.6	18.3 ± 5.8
T_max_ (h)	-	4.8 ± 3.5
C_0_ (ng/mL)	577.8 ± 121.7	-
C_max_ (ng/mL)	-	26.4 ± 4.6
AUC_last_ (ng·h/mL)	208.2 ± 23.6	550 ± 155.4
AUC_infinity_ (ng·h/mL)	219.2 ± 25.9	781.4 ± 311.6
F (%)	-	6.9 ± 1.8

* t_1/2_ obtained after i.v. injection was significantly different that from transdermal administration (one-way ANOVA, post hoc; Tukey’s test, *p* < 0.05).

**Table 4:** Average concentration and plasma-to-tissue partition coefficients (k_p_) of BP-3 in tissues determined under steady state conditions (n = 4, mean ± S.D.).

Tissues	Concentration (ng/mL or ng/g)	k_p_
Plasma	180.50 ± 52.29	-
Kidney	620.68 ± 297.70	4.00 ± 2.87
Liver	269.19 ± 52.14	1.54 ± 0.27
Spleen	155.71 ± 27.63	0.89 ± 0.13
Lung	710.00 ± 326.77	4.50 ± 2.87
Heart	363.14 ± 132.57	2.11 ± 0.87
Testis	136.74 ± 70.88	0.73 ± 0.16
Stomach	476.16 ± 99.72	2.70 ± 0.44
Small intestine	532.22 ± 296.66	2.92 ± 1.35
Large intestine	1174.44 ± 972.83	6.39 ± 5.77
Brain	352.75 ± 95.81	2.07 ± 0.81

## Data Availability

Not applicable.
